# Construction and demolition waste material library based on vision systems data

**DOI:** 10.1016/j.dib.2025.111927

**Published:** 2025-07-28

**Authors:** Maria Teresa Calcagni, Giovanni Salerno, Gloria Cosoli, Giuseppe Pandarese, Gian Marco Revel

**Affiliations:** a Department of Industrial Engineering and Mathematical Sciences, Università Politecnica delle Marche, Via Brecce Bianche 12, 60131 Ancona, Italy; b Department of Theoretical and Applied Sciences, eCampus University, Via Isimbardi, 10, 22060 Novedrate, Italy

**Keywords:** CDWs, Hyperspectral, Emissivity, Material spectral characterization, Vision-based measurement

## Abstract

The sustainable management of Construction and Demolition Wastes (CDWs) represents a crucial challenge for the European Union, considering that this wastes stream constitutes one of the main sources of man-made solid wastes. The implementation of strategies aimed at the recovery and recycling of these materials is essential to reduce the environmental impact of the construction sector and to foster the transition towards a circular economy model. However, one of the main obstacles for effective reuse and/or recycling of CDWs lies in the complexity of their composition, which includes a wide range of materials such as concrete, bricks, ceramics, metals, and wood, not rarely contaminated with harmful substances. In this context, this data article presents a comprehensive material library designed to collect, organise, and make available data from advanced material characterisation analyses based on vision systems data. Specifically, the library focuses on data obtained through two measurement techniques: infrared (IR) thermography and hyperspectral imaging (HSI). These methodologies were selected for their ability to provide complementary information on the chemical composition and physical properties of materials. The material library was developed as part of an in-depth study of CDW from building demolition and renovation operations in several EU countries. The data collection process included the preparation and analysis of representative samples, with the aim of ensuring maximum accuracy and reproducibility of the measurements. The data obtained were standardised and organised in a format compatible with the main statistical analysis and machine learning tools to facilitate their integration into predictive models and decision-making processes. The article describes in detail the library structure, data collection protocols, and practical applications in the fields of waste management and sustainable construction. In addition, the benefits of this resource for the scientific and industrial community are discussed, including the possibility of using the data to develop/fine-tune artificial intelligence (AI) algorithms capable of optimising sorting and recycling processes by recognition and discrimination among different types of CDW material using the aforementioned sensors. The material library represents a significant contribution to addressing the challenges posed by CDW management, promoting a more efficient use of resources and reducing the environmental impact of construction and demolition activities. This extensive database not only facilitates material characterisation and separation but also represents a solid basis for future technological innovation in the construction sector.

Specifications table

**Table:** 

Subject	Engineering
Specific subject area	Construction and demolition wastes (CDWs) analysis using hyperspectral imaging (HSI) and infrared (IR) thermography
Type of data	Raw Data for IR: .csv Processed data and results for IR data: .xlsx Raw Data for HS: .hdr, .dat Processed data for HS: .csv Images and results for HS data: .png
Data collection	The data were acquired by researchers from Università Politecnica delle Marche at their laboratories in Italy. The samples used for the analyses were sent by COMSA and Sorigué, two Spanish partners of the European project RECONSTRUCT (GA n. 101,082,265) who have CDWs collection sites (i.e., construction site and waste management site, respectively).
Data source location	Samples collection: COMSA construction site, Barcelona, Spain (41°27′00.7″N 2°10′50.6″E); Sorigué waste management site, Botarell (Tarragona), Spain (41°09′44.3″N 1°01′04.0"E).
Data accessibility	Repository name: Material library - Infrared and Hyperspectral dataset Data identification number: not available at present. Direct URL to data: https://zenodo.org/records/15976821 Instructions for accessing these data: click on the link, there is no password needed.
Related research article	ACTA IMEKO IR: G. Salerno, G. Cosoli, M. T. Calcagni, G. Pandarese, G.M. Revel, Uncertainty analysis in the estimation of construction and demolition wastes emissivity through infrared thermography, Acta IMEKO, 2025. DOI: https://doi.org/10.21014/actaimeko.v14i2.2056 METRO LIVENV HS: A. Mobili, M. T. Calcagni, G. M. Revel, J. Donnini, G. Cosoli, S. Sabbatini, F. Tittarelli, G. Salerno, E. Leoni, V. Corinaldesi, How to Quickly Characterize Construction and Demolition Wastes? Traditional and Advanced Portable Solutions in Comparison,2024 IEEE International Workshop on Metrology for Living Environment (MetroLivEnv). IEEE, 2024. p. 558–563. DOI: 10.1109/MetroLivEnv60384.2024.10615486

## 1 Value of the data

• These data are useful to analyse, recognize, and classify CDWs using IR thermography and HSI techniques.
• IR active (with specimen heating) can be exploited to evaluate the different behaviour of materials to received heat, especially by establishing a clear test protocol for the material emissivity estimation.
• HSI provides spectral fingerprinting characteristic of each sample; this allows comparison between materials and consequently classification, helping to distinguish different materials.
• Data fusion techniques may give more value, by seeing the material from different points of view, different material features can be extracted, and the techniques can complement each other, thus exploiting synergies between diverse sensing techniques.
• These data are very useful for researchers who want to further investigate these materials but do not have the material samples and data available.

## 2 Background

The raw data are provided for further investigation; replication of the tests is promoted for interesting comparison of the data itself and compatibility analysis. This work involved establishing a spectral and thermal database for demolition and construction waste, as these technologies were utilized for the investigation. Although a preliminary study had explored the background of these technologies' application, no comparable database was identified in the literature.

Material characterisation and classification of CDW are essential to improve recycling, limit landfill use and promote the sustainable use of natural resources. Knowing the composition of these materials helps to reintegrate them into the production cycle, reducing emissions and energy use and supporting a circular economy [1,2].

Digital technologies and artificial intelligence can automate and optimise these processes, reducing human effort and enabling faster and more accurate material selection [3,4]. Vision systems operating in different spectral ranges, such as infrared (IR) thermography and hyperspectral imaging (HSI), can identify materials, detect contaminants and guide decision-making, leading recycling more efficient and sustainable [5]. IR and HSI, in particular, reveal properties of materials that are invisible to the human eye, improving classification and recovery [[6], [7], [8], [9], [10]].

## 3 Data description

The dataset contains two main folders: ‘InfraRed’ and ‘HyperSpectral’, respectively containing the analysis data referred to in the title of the folder, the file ‘Materials_list.xlsx’ containing details of the sample labels, and the file ‘Read Me.docx’ containing information on the data structure.

The ‘Materials_list.xlsx’ file is a table containing the specimens information: sample number (list from 1 to the number of sample for each partner providing them, i.e., COMSA and Sorigué), material type, picture, and label; the labels are the names given to each specimen based on the name of the partner that has sent it (COM for COMSA and SOR for Sorigué), followed by the sample number and the number of the repeated test in the case of COMSA samples, and by a class abbreviation (e.g., ‘c’ stands for concrete, ‘b’ for brick, etc.), followed by the sample number (based on the number of samples tested for the considered class) and the number of the repeated test in the case of Sorigué samples; an example per partner is shown in Table 1 (first row for COMSA, second row for Sorigué).

**Table 1:** Examples of data contained in the file ‘Materials_list.xlsx’.

Sample number	Material	Picture	Label
1	Plastic		COM_1
1	Concrete		SOR_c1

For the raw data of both analyses, the names of the test folders are composed, in case of COMSA specimen, by the shortened name of the partner (COM), the sample number and the test number, e.g., COM_10_2 (tenth COMSA sample, second test). In case of Sorigué specimens, the tests were conducted with more than one specimen together, hence there is a subfolder per test with the name composed by the shortened name of the partner (SOR), the abbreviation of the class, and the number of test (1, 2, or 3), e.g., SOR_B_2 (brick class, second test); for one class (tiles and ceramic) the specimens were analysed on both sides, hence raw data are present for the ‘front’ (F) and ‘back’ (B) side, e.g., SOR_TC_F_1 and SOR_TC_B_1.

The ‘HyperSpectral’ folder contains the ‘Raw_data’ folder which contains data-cubes, the ‘ROI_data’ folder containing the selected ROIs (Region Of Interest), and ‘Data_images’ folder containing the resulting images.

• The **‘Raw_data’** folder in which there are the data-cubes of each acquisition for each sample. This folder contains the raw data for each sample. Each specimen has been recorded in three repetitions. Each subfolder is named after the corresponding material ‘+_ref’ that stands for reflectance data and includes:
  ○. dat files (raw data)
  ○. hdr files (configuration data)
  ○. png files (visible images of the sample during acquisition)
• The **‘ROI_data’** folder contains the reflectance spectra of the selected ROIs. It contains the spectra extracted from each data-cube by selecting the ROI that excludes the background. The spectra have been converted to .csv format to facilitate data processing.
• The **‘Data_images’** folder in which the spectra resulting from the processing are located. It contains visual representations of the spectra of each sample. These images display the spectra of the individual repeated tests together with the averaged spectrum and related standard deviation (i.e., confidence interval). All files are in .png format.
  The ‘InfraRed’ folder includes raw data in the ‘Raw_data’ folder, the results of each test grouped by class in the folder ‘Emissivity_values_per_class’, and the global results of the analysis in the file ‘Results.xlsx’.
• The **‘Raw_data’** is first divided into two subfolders (COM and SOR); within each of the two there are three folders for each specimen/group of specimens, each containing data from a single test, comprising 60 .csv files (one per frame acquired, as discussed in detail later).
• The folder **‘Emissivity_values_per_class’** contains a .xlsx file per class with the result of each single raw data (.csv file), thus 10 files per test. The .csv files are the raw data obtained from the thermal camera with a temperature value (measured by the camera) for each pixel. They were exported using Irbis 3 plus software (Infratec, Dresden, Germany, v. 3.0.0) and contain a header with general information (such as data size, temperature unit) and measured temperature data.
• The ‘Results.xlsx’ file summarizes the emissivity values for each material in terms of mean (Mean), minimum (Min), maximum (Max), and standard deviation (σ) values, and the number of measurements on which these values were obtained.

File structure information is summarized in the file ‘Read Me.docx’ for easy understanding of the data structure directly in the material library folder.

## 4 Experimental design, materials and methods

### 4.1 Hyper spectral analysis

#### 4.1.1 Description of instrumentation and measurement protocol for hyper spectral data

**Fig. 1:**
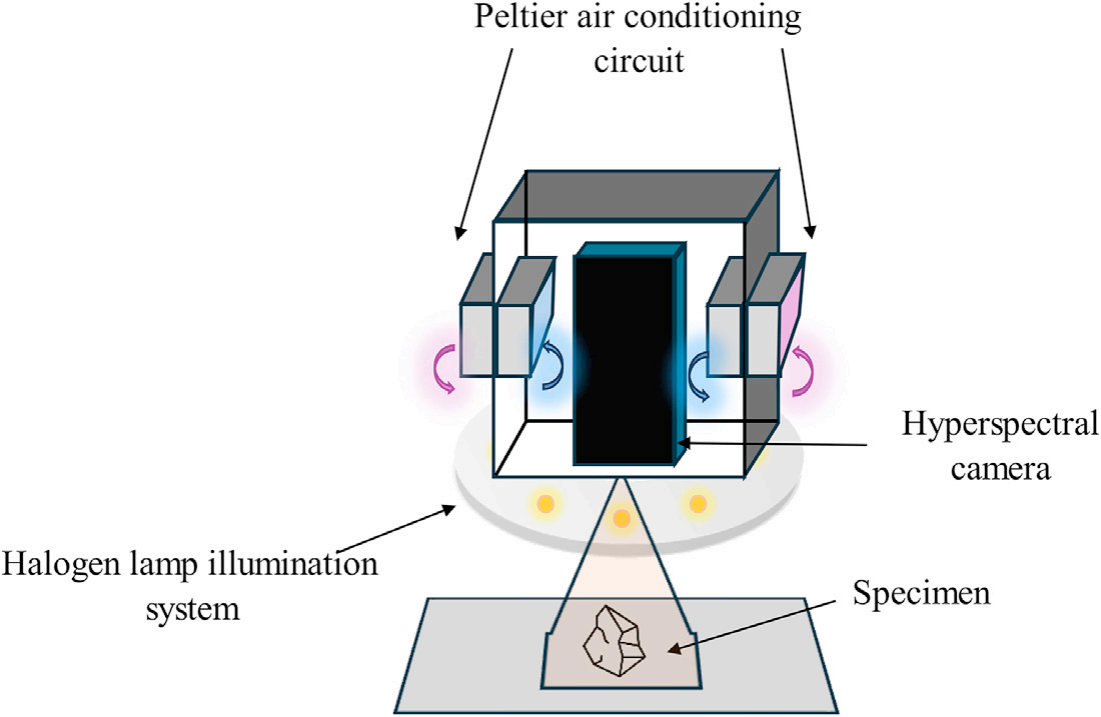
Hyperspectral camera acquisition system consisting of conditioning circuit and illumination system.

Measurement equipment comprised a hyperspectral camera fixed on a support that allowed its distance from the work surface to be varied, the black color of the latter to avoid light interference that could disturb the measurement. The bench consisted of i) a lighting system, i.e., halogen lamps connected to a power supply and arranged on a circular aluminium ring, ii) a conditioning system, and ii) a laptop computer with the Hinalea APP software (version 2.3.2 for Windows, Hinalea, USA, https://hinaleaimaging.com/contact/), connected to the camera and required for data management and acquisition (Fig. 1). The conditioning system, consisting of three Peltier modules and a thermocouple measuring the temperature inside the thermal insulation box, was purpose-built to maintain the camera in a temperature range of (20 ± 5) °C, in order to balance the temperature increase of the camera during the measurement, which led to the possibility of obtaining noisy results. The annular illumination system with eight 12-V halogen lamps was specially constructed to ensure uniform and stable illumination. Literature shows that halogen lamps maintain good spectral stability over time [11], covering near-infrared and visible wavelengths well, making them the best choice for this case study.

The hyperspectral camera performed the non-destructive analysis based on the acquisition of the datacube of each sample in terms of reflectance and the subsequent extrapolation of the spectra of the examined samples. The device used was the VIS-NIR 4250 camera from Hinalea, whose technical specifications are reported in Table 2 [12].

**Table 2:** Technical specification of 4250 VIS-NIR Hinalea.

*4250 VIS-NIR Hinalea – Technical specifications*	
*Dimension*	*197.7* mm *x 81* mm *x 78* mm
*Weight*	*1.25* kg
*Input Voltage*	*110* V *AC at 60* Hz*/ 220* V *AC at 50* Hz
*Working Temperature*	*(20±5)* °*C*
*Spatial sensor resolution*	*2.3 MP*
*Spectral Range*	*400 - 1000* nm
*Spectral Bands*	*300*
*Spectral Resolution*	*4* nm

The analysis stages for each sample are described in the following and the measurement chain is thoroughly explained.

1. **Sample and capture bench processing**.
  The test sample was chosen, wiped clean, and taken to the workbench. The black bench is cleansed to eliminate any possible residue left by the earlier sample; then, the new sample to be analysed was put on the measuring desk.
2. **Acquisition system setting up.**
  The VIS-NIR 4250 camera was plugged into the computer and switched on; the camera proprietary software, i.e., Hinalea APP, was launched.
3. **Calibrating black process***.*
  Black calibration was performed using the Hinalea APP, keeping the camera lens covered. This step was not repeated for each measurement, as the black is calibrated at the beginning of the acquisitions made on a single day.
4. **Air conditioning and lighting systems**.
  The cooling system was switched on to keep temperature always in the range (20 ± 5) °C. The halogen lamp system was switched on and it was plugged to power supply, which makes the lighting stable.
5. **Focusing and calibrating white.**
  The specimen to be analysed was brought into focus by setting the camera lens; then, the white target was placed at the same height as the specimen, thus maintaining the focus. An auto-exposure was then executed to adjust illumination and avoid over-saturating the image during the acquisition phase; white calibration is then carried out using the Hinalea APP. This step, unlike black calibration, must be repeated each time the focus of the sample under investigation changed.
6. **Sample acquisition process.**
  The blank target is removed and the sample to be acquired is placed under the camera; further self-exposure is made. Sample acquisition was performed by using the Hinalea APP; this stage took about 1–2 min. Three repeated acquisitions were made for each sample, in order to have more data to work with and to analyse the repeatability of the measurement.
7. **Recording data.**
  The data were collected by the native software, which outputs the hypercube; reflectance information was taken as the relevant data. The Hinalea APP also allows to select a ROI and export the reflectance spectrum of the selected region as a .txt file, which is much lighter and more manipulable than the hypercube.

**Fig. 2:**
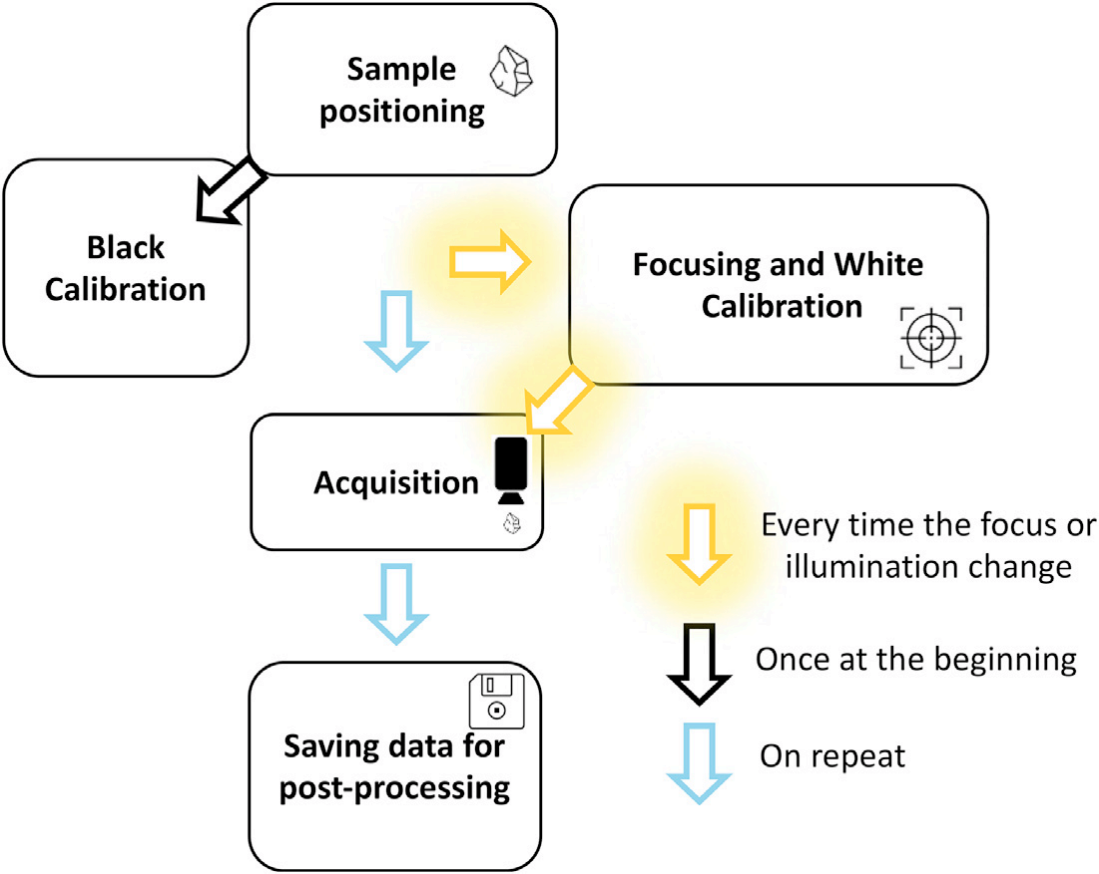
Measurement protocol for hyperspectral system followed during the tests.

A synthesis of the above explained measurement protocol is reported in Fig. 2. The data obtained from the hyperspectral camera measurements are processed by scripting a dedicated software using the Python programming language.

#### 4.1.2 Hyperspectral data processing

Initially, the previously acquired data were imported into the data processing code in order to calculate the mean and standard deviation values of the spectra of each sample; the maximum reflectance range is 0–1.5 [-] expressed as a normalised range according to the calibration process and the wavelengths have a range of 402–998 nm. Averages were computed by summing the reflectance values for each wavelength and dividing the total by the number of acquisitions of each sample (i.e., 3). The standard deviation was determined to establish the dispersion of the data around the mean, indicating the variability and repeatability of previous measurements for each sample. The individual sample results were considered in order to obtain a CDW library with the characteristic spectrum of each sample and its confidence interval. The following step is to obtain the average spectra and standard deviations of the seven classes considered in the study, i.e., plastic, concrete, brick, metal, wood, cardboard, ceramic and tile. The equations used to calculate the mean and standard deviation are given in Equation 1 and Equation 2, respectively. This was done in order to have a clear idea of the material characterization for each macro-class in terms of the variability of the spectra and, thus, to correctly interpret the results.

$$\begin{array}{c} {\bar{x}}_{\lambda }=\frac{1}{N}\sum_{i=1}^{N}x_{i,\lambda },\\ Equation\;1:Formula\;for\;mean\;spectrum\;calculation. \end{array}$$

Where:

• ${\bar{x}}_{\lambda }\;\left(-\right)$*is the mean spectrum*
• N*is the number of total samples considered for the computation of the mean spectrum*
• $x_{i,\lambda \;}\left(-\right)$*sample spectrum at index I*
  $$\begin{array}{c} \sigma_{\lambda }=\;\sqrt{\frac{1}{N-1}\sum_{i=1}^{N}{\left(x_{i,\lambda }-{\bar{x}}_{i,\lambda }\right)}^{2}}\\ Equation\;2:Formula\;for\;standard\;deviation\;calculation. \end{array}$$

Where:

• $\sigma_{\lambda }\;\left(-\right)$*is the standard deviation*
• N*is the number of total samples considered for the computation of the standard deviation*
• $x_{i,\lambda }\;\left(-\right)$*sample spectrum at index I*
• ${\bar{x}}_{i,\lambda }$(−)*is the mean spectrum at index I*

#### 4.1.3 Results

HSI-based analysis results in reflectance spectra for each material class and were included in the library. Specifically, the spectrum of each material was associated with the analysed sample; the average spectrum curve obtained during the three repeated acquisitions on each sample is indicated in red, and the confidence interval (*k* = 1) curves calculated over the three spectral acquisitions are reported in blue.

### 4.2 Infrared analysis

#### 4.2.1 Description of instrumentation and measurement protocol for infrared thermal data

**Fig. 3:**
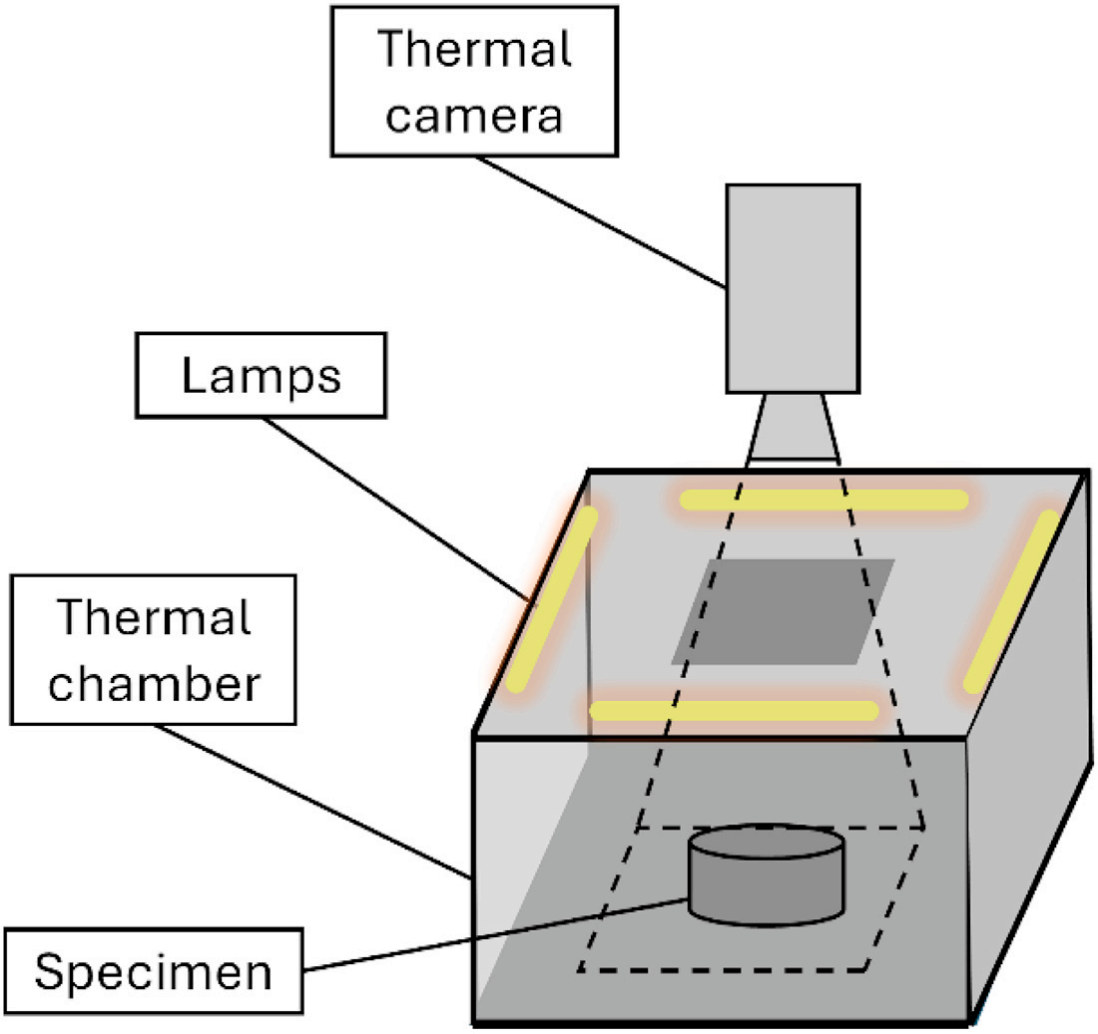
Setup used for the infrared acquisition.

The instrumentation used comprehended a thermal camera, installed on top of an isolating chamber, observing the internal part of the chamber through a hole on the top, and four halogen lamps used to heat the specimens during the acquisitions and placed on top of the internal part of the chamber, for a total power of 1100 W; an illustration of the setup is shown in Fig. 3.

The camera was installed 16 cm from the top of the chamber (distance from the lens), and 41 cm from the specimens.

The thermal camera used was the VarioCam 980HD (Infratec, Dresden, Germany [13]), whose main specifications are reported in Table 3.

**Table 3:** Technical specification of VarioCam 980HD.

*VarioCam 980HD – Technical specifications*	
*Dimension*	*190* mm *x 90* mm *x 94* mm
*Weight*	*1.15* kg
*Working Temperature*	*(−15 … 50)* °*C*
*Detector*	*Uncooled Microbolometer Focal Plane Array*
*Spatial sensor resolution*	*1024×768 pixels*
*Spectral Range*	*7500 - 14,000* nm
*Temperature measuring range*	*(−40 … 1200)* °*C*
*Measurement accuracy*	*± 1.5* °*C or ± 1.5 %*
*Temperature resolution at 30* °*C*	> 0.05 K

The acquisitions were performed using the software Irbis 3 plus (Infratec, Dresden, Germany, v. 3.0.0 [13]). The software supports the acquisition by controlling the thermal camera during the camera focusing, acquisition procedure, data saving and exporting in a suitable format (.csv in this case).

The test protocol, already explained in [14] involves the following steps:

1. **Specimen preparation.**
  Since the aim of the tests was to estimate the emissivity of the specimens, these were prepared by applying a paint or a tape with known emissivity values, and at least one portion of the specimen with the paint or tape must be present in each test. The paint used was the Aremco HiE-Coat-840-C (Valley Cottage, NY, United States [15]), which has a spectral emissivity value of ε = 0.89, considering a weighted average of emissivity in the analysed spectral range (7500 - 14,000 nm), with a measurement uncertainty of 0.01 (reported in terms of standard deviation, provided by the manufacturer). The paint required a curing step in an oven at 100 °C for an hour for proper adhesion. The tape was a standard known emissivity tape with an emissivity value of ε = 0.93, and it was an alternative in the cases the paint was not applicable, therefore for the specimens which cannot be subjected to the curing in the oven (e.g., some paper or plastic specimens).
2. **Camera focusing.**
  The software Irbis 3 plus can control the thermal camera, and it was used to set the camera focus.
3. **Specimens heating.**
  The halogen lamps were switched on to heat the specimens. The heating continued until a temperature rise of (10 ± 2) °C was reached, therefore the heating time was not the same for all the tests, depending on the heating rate of the specimens. The temperature rise had a variability of ± 2 °C due to the high heating rate of some specimens and to the fact that the lamps that continue to heat up even when were switched off due to their temperature. The temperature of the specimens was monitored through the software in real time by considering the average temperature of the specimen area with the paint or tape.
4. **Data acquisition and export**
  The acquisition was performed with a sequence of 60 frame acquired in 60 s (i.e., frame rate of 1 Hz), although only 10 frames of data were used (those after the lamps were switched off), to include all relevant data in the acquisition. The lamps were switched off right after the start of the acquisition to acquire a short warm-up and a long part of the cooling phase of the specimens. The software can also handle data storage and export.

**Fig. 4:**
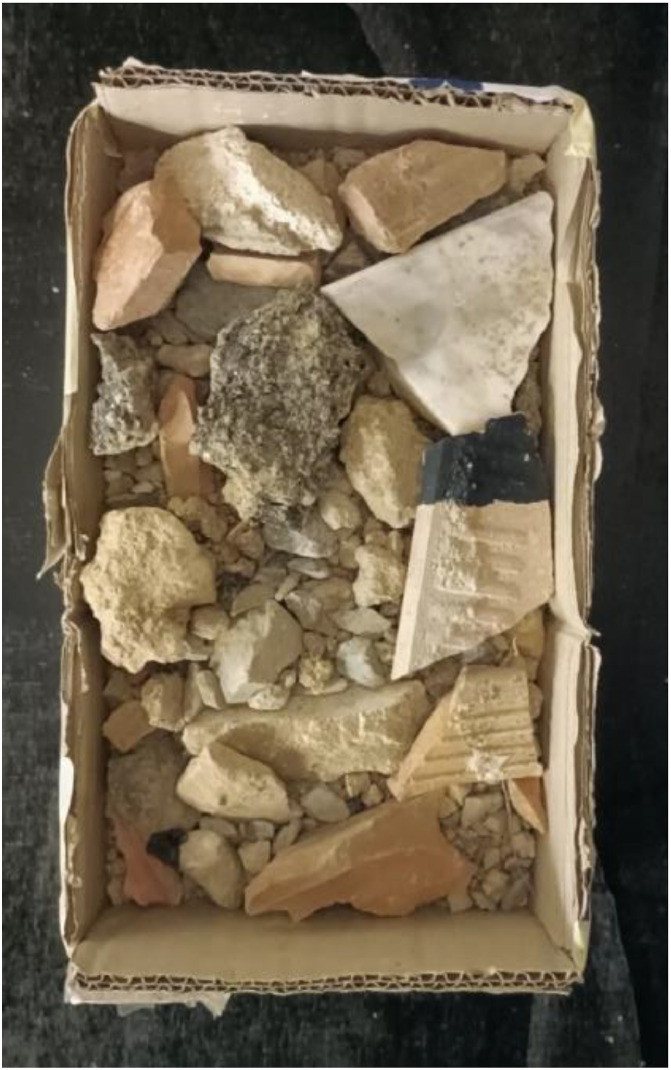
Mixed CDW specimen.

A slightly different approach was used for the mixed CDW class, since these specimens were composed of little stones, sand, pieces of concrete or tiles, and so on; the material was placed in a container (Fig. 4) for the acquisition. The test protocol was the same of the other specimen types, with the only difference of the shuffling of the specimens after each acquisition in order to comprehend the variability of this class by considering also the covered part of the previous acquisition in the next one. In this case the reference paint with known emissivity was applied on one of the bigger piece, that usually was a piece of concrete or tile (tile in Fig. 4), and this part was placed in the superior part to be clearly visible on the framed area.

#### 4.2.2 Data analysis

The thermal camera measures the radiance emitted by a surface, that, based on the Stefan-Boltzmann law, can be computed as:

$$\begin{array}{c} E=\;\sigma \;\varepsilon \;T^{4}\\ Equation\;3:Stefan-Boltzmann\;law. \end{array}$$

Where E (W/m^2^) is the radiance, σ the Stefan-Boltzmann constant (σ = 5.67×10–8 W/m^2^K^4^), ε (-) the emissivity, and T (K) the temperature. It is pivotal to underline that if the emissivity value estimated for a certain material and set on the camera software does not match the actual emissivity, the non-contact temperature measurement will be inaccurate. For this reason, and for an accurate measurement, the use of the paint (or tape) with known emissivity is fundamental. The estimation of the materials emissivity is based on the assumption that the specimen parts with and without the paint (or tape), respectively the reference and test part, are in thermal equilibrium (i.e., at the same temperature). By setting the reference emissivity value in the software, the camera can accurately measure the temperature in the reference area, but the temperature measured in the test area will appear different due to the mismatch in the emissivity. Considering this observation, it is possible to write:

$$\begin{array}{c} E_{test}=\;\sigma \;\varepsilon_{test}\;T_{test}^{4}=\;\sigma \;\varepsilon_{ref}\;\;T_{test,meas}^{4}\\ Equation\;4:Radiant\;exitance\;of\;the\;specimens,\;expressed\;asactual\;and\;measured\;values. \end{array}$$

Where:

• *E_test_ is the radiant exitance of the specimens, measured by the thermal camera,*
• ε*_test_ is the test material emissivity (unknown),*
• ε*_ref_ is the material reference emissivity,*
• *T_test_ is the actual test temperature, which is equal to reference temperature,*
• *T_test, meas_ is the measured test temperature.*

With the assumption of thermal equilibrium, Equation 4 can be rewritten as:

$$\begin{array}{c} \varepsilon_{test}={\left(\frac{T_{test,meas}\;\left[K\right]}{T_{ref}\;\left[K\right]}\right)}^{4}\varepsilon_{ref}\\ \mathrm{Equation}\;\mathrm{5:}\;\mathrm{Specimen}\;\mathrm{emissivity}\;\mathrm{computation}\;\mathrm{based}\;\mathrm{on}\;\mathrm{its}\;\mathrm{temperature}\;\mathrm{and}\;\mathrm{reference}\;\mathrm{emissivity}\\ \mathrm{and}\;\mathrm{temperature.} \end{array}$$

**Fig. 5:**
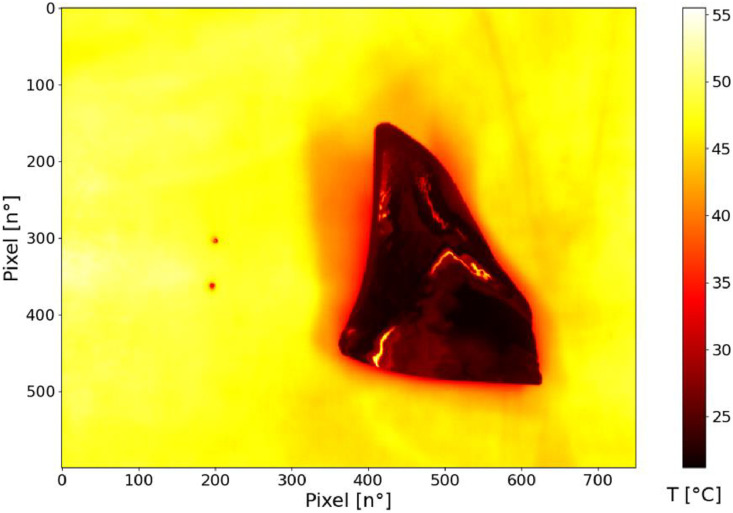
Example of thermal image acquired.

**Fig. 6:**
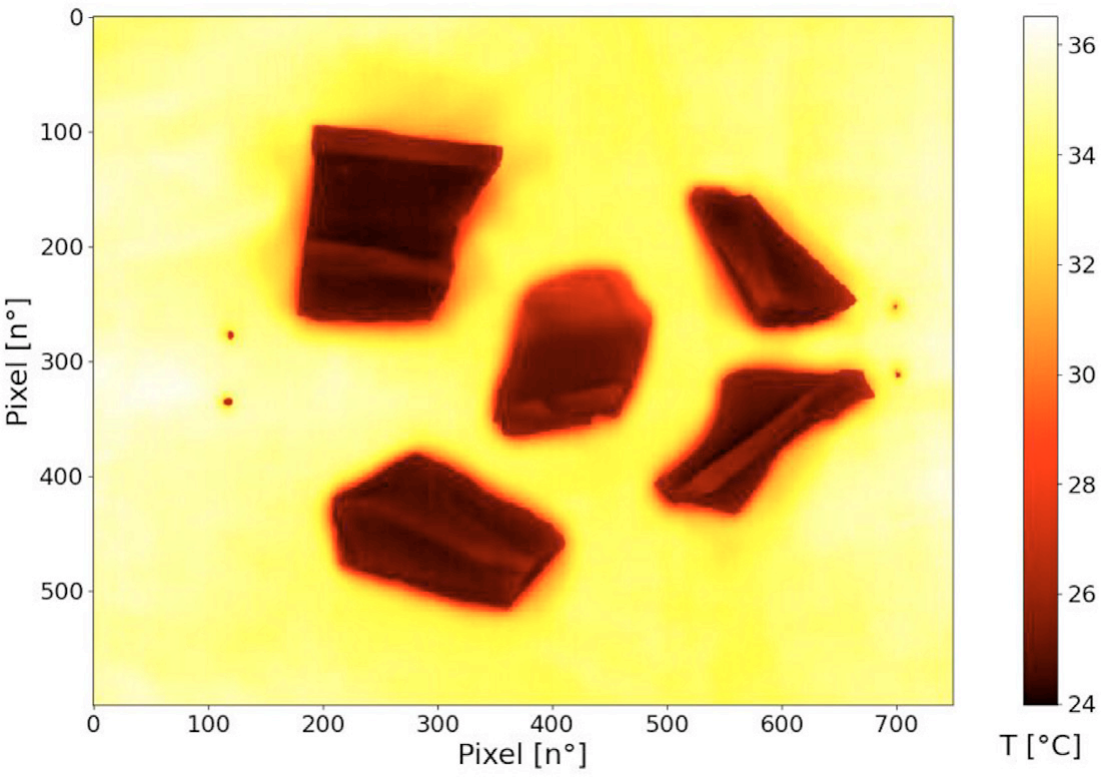
Example of thermal image acquired on a group of specimens.

The exported .csv data were analysed using Python programming language to extract two ROIs per specimen per test, one for the reference and one for the test part (examples in Fig. 5, Fig. 6). In some cases, more than one specimen was analysed in the same test (Fig. 6); the reference paint (or tape) was applied only in one of them, basing on the similarities in shape and dimension, it was assumed that the temperatures are equal. The mean temperature is extracted from the selected ROI, and the emissivity value was obtained using the Equation 5.

#### 4.2.3 Results

Each specimen was analysed three times, and the results were averaged; finally, the results were averaged for each class. Hence, both intra- and inter-specimen variability was taken into account.

For the mixed CDW class, each little ROI was considered as a specimen in order to properly reflect the variability of this class; finally, also for this class, the results were obtained averaging the results obtained on all the specimens.

For each class, the results were summarized in the file ‘Results.xlsx’ in the ‘InfraRed’ folder, including mean, minimum, maximum, and standard deviation values.

## Limitations

Some complex issues that were addressed during the analyses are listed below:

• Small number of samples: not all material classes had a satisfactory number of samples. In particular, the information about the materials was not satisfactory to frame them into the macro-classes and identify them through spectral and thermal characteristics. Hence, ambiguous samples were excluded from the work.
• Tests were limited to the specimens provided by the project partners and the classes of interest were agreed with the project stakeholders. More detailed analyses on subclasses were beyond the scope of the study.
• Some materials had inhomogeneities or reflective surfaces; this resulted in considerable difficulties in analyzing some samples. For this reason, pictures saturated areas were excluded.
• Illumination and conditioning were important aspects to consider in the study of the samples, as far as the hyperspectral camera is concerned, and the establishment of such systems was critical to the success of the analysis.

## Ethics statement

The authors have read and follow the ethical requirements for publication in Data in Brief and confirm that the current work does not involve human subjects, animal experiments, or any data collected from social media platforms.

## Credit author statement

**Maria Teresa Calcagni:** Conceptualization, Data curation, Formal analysis, Investigation, Methodology, Software, Writing – original draft. **Giovanni Salerno:** Conceptualization, Data curation, Formal analysis, Investigation, Methodology, Software, Writing – original draft. **Gloria Cosoli:** Conceptualization, Methodology, Project administration, Supervision, Writing – review and editing. **Giuseppe Pandarese:** Conceptualization, Investigation, Methodology, Writing – review and editing. **Gian Marco Revel:** Conceptualization, Funding acquisition, Project administration, Supervision, Writing – review and editing.

## Data Availability

ZenodoMaterial library - Infrared and Hyperspectral dataset (Original data)
